# Biochemical and Structural Properties of a Thermostable Mercuric Ion Reductase from *Metallosphaera sedula*

**DOI:** 10.3389/fbioe.2015.00097

**Published:** 2015-07-13

**Authors:** Jacob H. Artz, Spencer N. White, Oleg A. Zadvornyy, Corey J. Fugate, Danny Hicks, George H. Gauss, Matthew C. Posewitz, Eric S. Boyd, John W. Peters

**Affiliations:** ^1^Department of Chemistry and Biochemistry, Montana State University, Bozeman, MT, USA; ^2^Department of Chemistry and Geochemistry, Colorado School of Mines, Golden, CO, USA; ^3^Department of Microbiology and Immunology, Montana State University, Bozeman, MT, USA; ^4^Thermal Biology Institute, Montana State University, Bozeman, MT, USA

**Keywords:** mercuric reductase, mercury detoxification, thermophile, thermostability, structure, biosensor, MerA

## Abstract

Mercuric ion reductase (MerA), a mercury detoxification enzyme, has been tuned by evolution to have high specificity for mercuric ions (Hg^2+^) and to catalyze their reduction to a more volatile, less toxic elemental form. Here, we present a biochemical and structural characterization of MerA from the thermophilic crenarchaeon *Metallosphaera sedula*. MerA from *M. sedula* is a thermostable enzyme, and remains active after extended incubation at 97°C. At 37°C, the NADPH oxidation-linked Hg^2+^ reduction specific activity was found to be 1.9 μmol/min⋅mg, increasing to 3.1 μmol/min⋅mg at 70°C. *M. sedula* MerA crystals were obtained and the structure was solved to 1.6 Å, representing the first solved crystal structure of a thermophilic MerA. Comparison of both the crystal structure and amino acid sequence of MerA from *M. sedula* to mesophillic counterparts provides new insights into the structural determinants that underpin the thermal stability of the enzyme.

## Introduction

The ionic form of mercury, which is one of the most toxic metals known to biology (Gertrud et al., 1989; Nies, 2003; Vetriani et al., 2005), is naturally present at elevated concentrations in many hydrothermal vents, hot springs, and acid mine drainage fluids (Batten and Scow, 2003; Simbahan et al., 2005; Vetriani et al., 2005; King et al., 2006; Boyd et al., 2009; Wang et al., 2011). In these environments, biology utilizes a finely tuned protein catalyst termed the mercuric reductase (MerA) (encoded by the *merA* gene) in order to reduce toxic ionic mercury (Hg^2+^) to the far less toxic, volatile, and elemental form (Hg^0^). The reaction catalyzed by MerA follows the reaction scheme of NADPH + Hg^2+^ → NADP^+^ + Hg^0^ (Barkay et al., 2003). MerAs, which are part of the disulfide oxidoreductase (DSOR) family (Fox and Walsh, 1982), are ancient enzymes, having arisen in high temperature environments after the great oxidation event ~2.4 billion years ago (Barkay et al., 2010). Since that time, evolution has finely tuned MerA through recruitment of regulatory and transport proteins (Boyd and Barkay, 2012) to serve a diversity of organisms, including both Archaea and Bacteria, which encounter Hg^2+^ ions in less extreme mesophilic settings, while retaining extremely high stability and substrate specificity. These characteristics of mercuric reductases lend them to possible sensor applications, wherein the redox properties of the enzyme could be coupled to an amplifiable electrical signal (Adami et al., 1995; Han et al., 2001; Zhang et al., 2011). A stable mercuric reductase may also be used to potentially mitigate mercury contamination (Nascimento and Chartone-Souza, 2003).

*Metallosphaera sedula* (*Mse*), isolated previously from Pisciarelli Solfatara in Naples, Italy (Gertrud et al., 1989), has a minimum and maximum temperature for growth range of 50–80°C (Auernik et al., 2008a). Pisciarelli Solfatara itself contains a variety of thermal features that range in temperature from ~30°C to nearly 100°C, and a pH range of 1.5 to around 6.0 with elevated concentrations of heavy metals, including Hg^2+^ at concentrations up to 0.005 g/kg (Huber et al., 2000). The genome sequence of *Mse* was completed in 2008, (Auernik et al., 2008b), making it possible to identify mechanisms of Hg^2+^ tolerance at the genomic level. The *mer* operon in *Mse* includes both MerA and MerH, where MerH may aid metal trafficking to the MerR transcription factor (Schelert et al., 2013).

A variety of MerAs have been characterized previously, most notably a protein encoded on a transposon isolated from *Pseudomonas aeruginosa*, which is termed Tn*501* (Fox and Walsh, 1982), as well as MerA from *Bacillus cereus* (*Bc*MerA) (Schiering et al., 1991) and a MerA from a deep brine environment, termed ATII-LCL (Sayed et al., 2013). Collectively, these biochemical studies have revealed MerAs that exhibit *K*_m_ values for Hg^2+^ that range from 9–70 μM and specific activities that range from 1.05–50 μmol/min⋅mg. Structural characterization was first carried out on *Bc*MerA (Schiering et al., 1991) and later on Tn*501* (Ledwidge et al., 2005). Most recently, the Tn*501* structure has been solved in complex with Hg^2+^(Lian et al., 2014). Structural characterization confirmed that MerA is a member of the DSOR protein family, which adopts a βαββαβ fold, and which is known to catalyze pyridine-dependent substrate reduction with a characteristic active site CXXXXC motif (Argyrou and Blanchard, 2004). Some MerAs also harbor an additional N-terminal GMTCXXC motif (Boyd and Barkay, 2012) that assists in metal recruitment (Ledwidge et al., 2005). A third pair of conserved cysteines are located in a flexible region on the C-terminal domain, and are responsible for delivering mercuric ions to the active site of the opposing monomer (Lian et al., 2014).

Despite these advances, the structural characterization of a MerA from a thermophile has yet to be conducted, even though this is critical for understanding the properties of enzymes involved in mercury detoxification of high-temperature environments where mercury concentrations are very high. Structural characterization is important for both understanding the thermophilic origins of the protein (Barkay et al., 2010; Boyd and Barkay, 2012) as well as for possible incorporation into stable biotechnologies. Here, we report biochemical and structural characterization of a thermostable MerA from the aerobic thermoacidophilic Crenarchaeon *Mse* (*Mse*MerA).

## Materials and Methods

### Bioinformatics

MerA homologs were compiled from the Department of Energy-Integrated Microbial Genomes database using BLASTp and the *Tn501* MerA as a query. Representative homologs were screened for conserved residues that define MerA (as described above), and those protein sequences with these residues were aligned using CLUSTALX (version 2.0.8) specifying the Gonnet 250 protein substitution matrix and default gap extension and opening penalties (Larkin et al., 2007), with dihydrolipoamide dehydrogenase from *Magnetospirillum magneticum* AMB-1 (YP_423326), *Thermus thermophilus* HB27 (YP_005669), and *Pseudomonas fluorescens* Pf0-1 (YP_351398) serving as outgroups. N-terminal “NmerA” sequence was trimmed from the alignment block as previously described (Barkay et al., 2010) and the phylogeny of MerA was evaluated with PhyML (ver. 3.0.1) (Guindon et al., 2010) using the LG amino acid substitution matrix with a discrete four category gamma substitution model and a defined proportion of invariant sites. A consensus phylogenetic tree was projected from 100 bootstrap replications using FigTree (ver. 1.2.2) (http://tree.bio.ed.ac.uk/software/figtree/).

Structural superimpositions were generated by the program UCSF Chimera (Pettersen et al., 2004). The protein sequence of *Mse*MerA was blasted with NCBI BLASTp. The top eight hits were compared with mesophilic mercuric reductases from *Staphylococcus aureus*, *B. cereus*, *P. aeruginosa*, and a sequence from a hydrothermal deep-sea brine environment, ATII-LCL (Sayed et al., 2013). It should be noted that while the ATII-LCL sequence was isolated from a hydrothermal vent system with a temperature of 68°C, the optimum temperature for activity was shown to be 30–50°C (Sayed et al., 2013), indicating that it is not adapted to the thermal regime from where it was isolated or that the environment from where it was isolated is variable with respect to temperature. VADAR was used to evaluate the surface area and charged residue percentage of MerA homologs (Willard et al., 2003), while the ProtParam tool available from ExPASy was used to calculate the aliphatic index of MerA homologs (Gasteiger et al., 2005).

### Expression and purification

*Mse*MerA DSM 5348 sequence was codon-optimized and synthesized by GenScript USA Inc. with an N-terminal 6× His-tag (Data Sheet 1 in Supplementary Material). The gene was cloned into MCS1 of pETDuet-1 and transformed into *Escherichia coli* BL21DE3 cells (Novagen, EMD Millipore, USA). Sequence-based confirmation of *Mse*MerA transformation was performed by Davis Sequencing, Inc. (1450 Drew Ave, Suite 100, Davis, CA, USA).

Fifty milliliters of Luria-Bertani (LB) broth, supplemented with 0.5 mM riboflavin and 0.1 g/L ampicillin, were inoculated with recombinant *E. coli* cells containing *Mse*MerA and shaken at 250 rpm at room temperature overnight. One liter of LB medium, as described above, was inoculated with 2 mL from the overnight culture, and shaken at 250 rpm until an OD_600_ of 0.5–0.7 was reached. About 2 mM IPTG was added and expression was carried out for 4 h, after which the cultures were centrifuged at 6000 × *g* for 10 min (4°C), with the resultant cell pellet immediately being flash frozen in liquid nitrogen and stored at −80°C. Each liter of cell culture yielded 3.0–3.5 g of cell paste.

Cell paste was subjected to three freeze/thaw cycles to facilitate lysis, after which cells were re-suspended in 5 mL Buffer A (100 mM NaCl, 50 mM MOPS with a pH of 6.7, 25 mM imidazole) per gram of cells. Lysozyme and deoxyribonuclease (DNase) were added to final concentrations of 0.1 mg/mL along with phenylmethylsulfonyl fluoride (PMSF)-saturated isopropanol to a final concentration of 0.1% v/v, and this mixture was incubated for 30 min at room temperature. Triton X-100 was then added to a final concentration of 1% v/v, and this was mixed for 30 min. The crude lysate was then clarified by centrifugation at 100,000 × *g* for 1 h (4°C). The resulting clarified lysate was observed to have a yellow color.

Purification of *Mse*MerA was carried out using a 75 mL gradient from 100% Buffer A to 100% Buffer B (100 mL NaCl, 50 mM MOPS with a pH 6.7, 250 mM imidazole) on a 2 mL Ni-NTA column (Qiagen) at 3 mL/min. Seven milliliter fractions were collected and further analyzed with an SDS-PAGE gel. Fractions containing pure protein were combined and concentrated to 10 mg/mL, buffer exchanged to Buffer C (10 mM MOPS pH of 6.7), and the protein was then concentrated to 30 mg/mL and flash-frozen in liquid nitrogen. Purity of the protein was confirmed by SDS- and Native PAGE (Figure S1 in Supplementary Material). A yield of 1.5 mg of pure protein per liter of growth culture was achieved.

### Activity assay

Activity assays were carried out in 100 mM NaCl, 50 mM MOPS with a pH of 6.7, 0.2 mg/mL *Mse*MerA, and 1 mM HgCl_2_, and these were initiated by the addition of 0.2 mM NADPH, similar to previously established procedures (Fox and Walsh, 1982). For kinetic studies, the concentration of Hg^2+^ ranged from 28.6 μM to 2.77 mM. NADPH oxidation was monitored at 338 nm using a Cary 6000 UV/Vis spectrometer equipped with a 1 × 1 Peltier. Assays were conducted from 37 to 70°C, above which temperature the rate of non-enzymatic NADPH oxidation was too high to accurately measure enzymatic activity. In order to determine the thermostability of *Mse*MerA, an aliquot of the enzyme was assayed at 37°C and the remaining protein was boiled at 97°C for 100 min, after which the enzymatic activity was once again measured at 37°C.

### Crystallization and structure determination

*Mse*MerA crystals were obtained using the hanging drop method. Crystallization drops contained 0.085M TRIS (pH 8.5), 15% v/v glycerol, 14% w/v PEG400, 0.19M LiSO_4,_ and 20 mg/mL protein. Crystals were obtained after 2 weeks, mounted on cryo loops, and shipped to the Stanford Synchrotron Radiation Lightsource for X-ray data collection. Diffraction data were collected at 100 K using the 12-2 beamline. Diffraction images were indexed, integrated, and scaled using HKL2000 (Otwinowski and Minor, 1997).

The structure of *Mse*MerA was solved to 1.6 Å using CCP4 molecular replacement (Cowtan et al., 2011) of Tn*501*MerA (PDB ID: 1ZK7), which shares 37% amino acid identity with *Mse*MerA. Model building was performed in Coot (Emsley et al., 2010) and coordinates were refined to reasonable stereochemistry at a resolution 1.6 Å (Figure S3 in Supplementary Material) using REFMAC5 (Murshudov et al., 1997). The structure was validated using MolProbity (Chen et al., 2010) and all molecular images were calculated in PyMol (Delano, 2002). Structural superimpositions were generated both with 1ZK7 (Ledwidge et al., 2005) and 4K7Z (Lian et al., 2014), in which the active site cysteines were substituted by alanines and could be solved in complex with the Hg^2+^ ion.

## Results

### Thermal adaptation of *Mse*MerA

**Figure 1:**
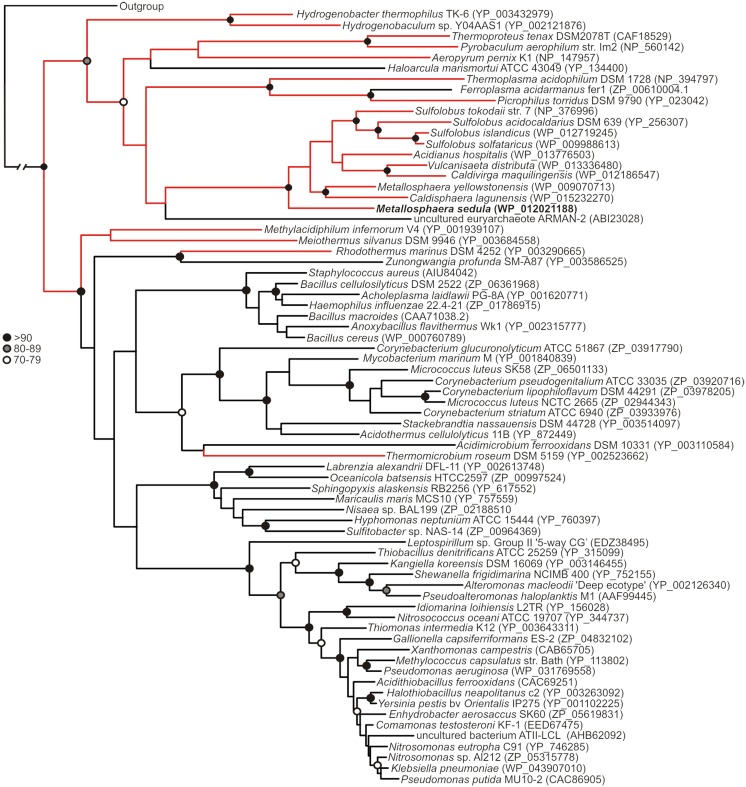
**Maximum-likelihood phylogenetic reconstruction of MerAs, with homologs from thermophilic taxa highlighted in red**. *Mse*MerA is boldfaced. Bootstrap support is indicated by black (>90), gray (80–89), and open (70–79) circles. Nodes with no symbol exhibited bootstrap values of <70.

**Figure 2:**
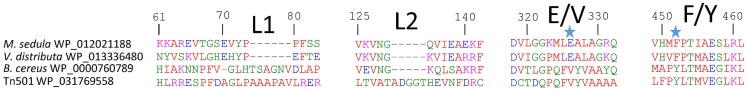
***Mse*MerA aligned with other MerAs reveals two loop regions, L1 and L2, which may be involved in conferring thermostability, and two positions at 326 and 452 (highlighted with stars), where the active site region is different between thermophiles and mesophiles**.

Phylogenetic reconstruction of representative core (NmerA trimmed) MerA sequences revealed a number of deeply branching lineages from thermophilic taxa, consistent with previous analyses that indicate MerA likely originated in a high temperature environment (Schelert et al., 2004; Barkay et al., 2010; Boyd and Barkay, 2012). *Mse*MerA clustered among MerA from thermophilic crenarchaeota (Figure 1). Sequence alignments reveal both the active site CXXXXC motif and C-terminal cysteines that are conserved among all MerA sequences. However, several key differences were observed that may be involved in conferring thermotolerance (Figure 2). Specifically, the thermophilic enzymes are missing regions corresponding to amino acids 66–71 and 130–134 Tn*501* (Tn*501*MerA numbering), suggesting a reduction in loop regions in comparison to the mesophilic enzymes (Figure 2). Two sets of residues, V317 and Y441, are within putative coordination distance of the active-site mercury. These residues are substituted for an E and F, respectively, in *Mse*MerA and other thermophiles with the exception of *Hydrogenobacter thermophilus* TK-6 (YNP_003432979) and *Hydrogenobaculum* sp. Y04AAS1 (YNP_002121876).

**Figure 3:**
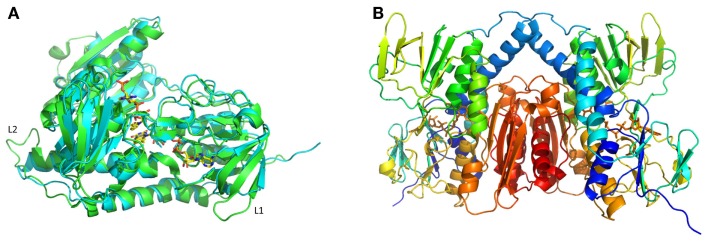
**(A)** Structural superimposition of *Mse*MerA monomer (cyan) with Tn*501*MerA (green) reveals a decrease in loop regions (labeled L1 and L2) in *Mse*MerA. **(B)** Cartoon representation of a dimer of *Mse*MerA with bound FAD.

A comparison of the *Mse*MerA crystal structure to the previously determined Tn*501*MerA structure (PDB: 1ZK7) (Ledwidge et al., 2005) reveals that the two structures are highly similar, with an overall C-alpha deviation of 1.5 Å rmsd as calculated by Dali Lite (McWilliam et al., 2013). Two particular loop regions are shorter in *Mse*MerA (Figure 3A). This was further supported by VADAR (Willard et al., 2003), which calculated a 4% decrease in coil regions in *Mse*MerA. The calculated surface area of *Mse*MerA, 19,966.5 Å^2^, is slightly reduced in comparison to Tn*501*MerA, with a surface area of 21,217.4 Å^2^.

MEGA (Tamura et al., 2013) was used to compile an amino acid composition chart for the sequences examined. The thermophiles were observed to have a larger number of positively charged amino acids. VADAR calculated the total charged residues in *Mse*MerA to be 25% of residues compared to 21% of residues in *Tn501*MerA, and 24% in *Bc*MerA. An increase in ionic interactions may therefore represent a factor contributing to MerAs thermal stability (Szilágyi and Závodszky, 2000). The aliphatic index of *Mse*, Tn*501*, and *Bc* MerAs were calculated by ExPASy’s ProtParam tool (Gasteiger et al., 2005), and found to be 101.63, 98.65, and 97.86, respectively, again in agreement with *Mse*MerA having higher thermostability (Ikai, 1980).

### Biochemical characterization

**Figure 4:**
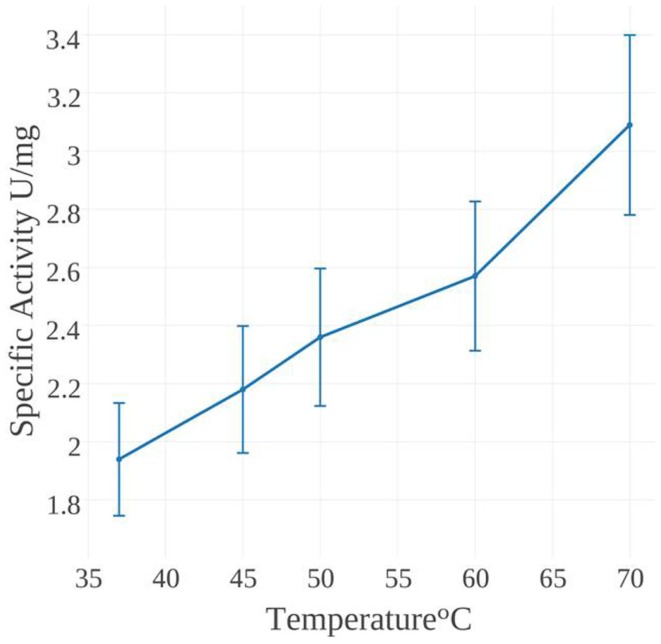
**NADPH oxidation activity of *Mse*MerA incubated at temperatures ranging from 37 to 70°C**.

The specific activity of *Mse*MerA was examined from 37 to 70°C (Figure 4). One unit of activity was defined as 1 μmol NADPH oxidized per minute. At 37°C, the specific activity was found to be 1.9 U/mg, increasing up to 3.1 U/mg at 70°C. Mercury dependence of *Mse*MerA was determined, with *K*_m_ values of 400 and 150 μM at 37 and 70°C, respectively. Specific activity was not determined above 70°C due to the difficulty of discriminating between enzymatic and non-enzymatic NADPH oxidation at high temperatures. The thermal stability of *Mse*MerA was tested by incubating the enzyme at 97°C for up to 100 min, followed by assessment of enzymatic activity at 37°C. Even after 100 min of incubation at 97°C, no decrease in overall activity was observed when compared to the untreated enzyme (Figure S2 in Supplementary Material). The *K*_cat_ at 70°C was found to be 23 s^−1^, with a *K*_cat_/*K*_m_ of 0.15 μM^−1^ s^−1^.

### Structural characterization of *Mse*MerA

*Mse*MerA crystals were obtained using vapor diffusion in a precipitating solution of 14% polyethylene glycol 4000 and 0.19M lithium sulfate. These crystals belonged to space group P22_1_2_1_ and contained two monomers per asymmetric unit, assembled into one homodimer (Figure 3B). The crystal structure of *Mse*MerA was solved to 1.6 Å, with *R* and *R*_free_ values of 16.9 and 19.6%, respectively. Bound FAD was observed, suggesting that these molecules act to stabilize the structure. No mercury was observed in the active site. As expected based on the sequence alignment, a clear reduction in loop regions was observed in comparison to Tn*501*MerA (Figure 3A). No electron density for the carboxy terminus of *Mse*MerA was identified from 440 to 448, including the conserved pair of cysteines at residues 446 and 447. This is in agreement with the carboxy terminus being able to undergo conformational changes during the catalytic cycle (Lian et al., 2014). The solved structure has been deposited in the Protein Data Bank with the accession code 4YWO.

## Discussion

Bioinformatic and phylogenetic data overwhelmingly support *Mse*MerA being a thermostable protein, as illustrated by features consistent with other enzymes from thermophiles, including a reduction in loop regions, a greater percent of charged amino acids, and an overall reduced surface area in comparison to its mesophilic counterpart. Collectively, these strategies are likely to interact synergistically to convey the high degree of thermostability observed. Retention of 100% activity after incubation at 97°C for 100 min further confirms the highly thermostable nature of *Mse*MerA.

Though practical constraints made measuring specific activity above 70°C impossible, catalytic activity was found to increase over the range of 37–70°C, with a *V*_max_ of 3.1 U/mg at 70°C. This places *Mse*MerA in the range of average activity when compared to other MerAs (Table 1). The *K*_m_ for Hg^2+^of *Mse*MerA was found to decrease from 400 μM at 37°C to 150 μM at 70°C, indicating a higher affinity for Hg^2+^ ions at elevated temperatures. The *K*_m_ of *Mse*MerA is around an order of magnitude higher than that found for other MerAs (Table 1), and may be an adaptive strategy to cope with elevated Hg^2+^ concentrations commonly encountered in the acidic, high temperature environments where *Mse* resides (King et al., 2006; Boyd et al., 2009; Wang et al., 2011).

**Table 1:** **MerA comparison**.

	Optimum growth temperature (°C)	Optimum temperature for enzyme activity (°C)	*K*_m_ (μM)		Specific activity (U/mg)	Amino acid substitution at the position V/Y 317/441 (Tn *501* numbering)	Reference
			Hg	NADPH	
*M. sedula*	50–79	>70	400^a^/150^b^	ND*	1.9^a^/3.1^b^	E/F	This work
*Pa*Tn*501*	25–42	55–65	12	6	12.7	V/Y	Fox and Walsh (1982)
ATII-LCL	~68	30–50	8.65	4.35	50	V/Y	Sayed et al. (2013)
*Azotobacter Chroococcum*	26	45	11.11	ND	25	ND	Ghosh et al. (1999)
*Klebsiella pneumoniae*	37	40	75	ND	9	V/Y	Zeroual et al. (2003)
*B. cereus*	37	ND	30	ND	ND	V/Y	Rennex et al. (1994)
*E. coli R831*	37	ND	13	6	1.05	ND	Schottel (1978)

*ND, not determined.

*^a^Measured at 30°C*.

*^b^Measured at 70°C*.

The *K*_cat_ of *Mse*MerA is 23 s^−1^, which is very similar to the *K*_cat_ of ATII-LCL at 22.5 s^−1^ (Sayed et al., 2013) and also similar to *Bc*MerA at 12 s^−1^ (Rennex et al., 1994). The higher *K*_m_ value observed in *Mse*MerA translates to the lowest overall catalytic efficiency, with a *K*_cat_/*K*_m_ of 0.15 μM^−1^ s^−1^.

Though *P. aeruginosa* (*Pa*) from which the *Tn501* transposon was isolated is a mesophilic organism, the MerA enzyme was found to have optimal activity at 55–65°C, and retained full activity at 37°C even following a 10-min incubation at 100°C (Nakahara et al., 1985; Vetriani et al., 2005). Intriguingly, phylogenetic analysis indicates that *Tn501*MerA groups closely with the mesophiles (Figure 1). Conversely, phylogenetic analysis of MerA from a high temperature brine pool, ATII-LCL (Sayed et al., 2013), was found to group with MerA sequences from mesophilic organisms (Figure 1). While the environment from which ATII-LCL was isolated is at 68°C, the enzyme has maximum activity over a range of 30–50°C and, when measured at 37°C, was found to be half inactivated after a 10-min incubation at 75°C (Sayed et al., 2013). The ATII-LCLMerA is therefore not nearly as thermostable as *Mse*MerA, and is not adapted to its local environment, with respect to the thermal regime, but is adapted with respect to salinity regime.

**Figure 5:**
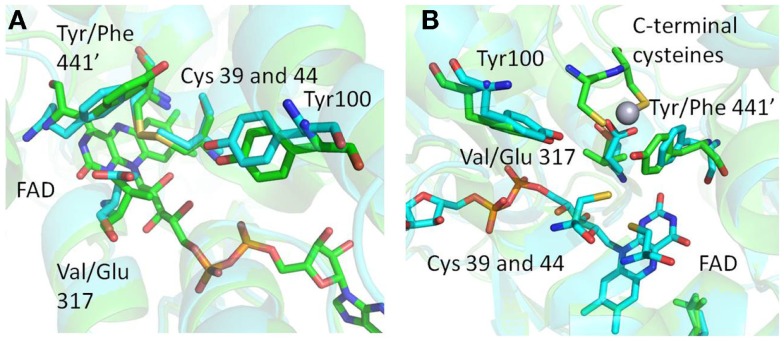
**(A)** Structural superimposition of *Mse*MerA with Tn*501*MerA 1ZK7 shows the Y441′/V317 amino acids conserved in mesophiles and the F441/E317 amino acids conserved in thermophiles, suggesting an alternative Hg^2+^ coordination strategy in *Mse*MerA. **(B)** An alternative angle of the active site environment of *Mse*MerA superimposed onto Tn*501*MerA 4K7Z, which depicts the Hg^2+^ ion bound to the c-terminal cysteines. The monomer with the C-terminal cysteines is noted by a “prime” designation.

The structure of *Mse*MerA reveals a dimeric biological assembly, as has been shown with previous structures (Schiering et al., 1991; Ledwidge et al., 2005; Lian et al., 2014). With this architecture, the active site cleft on one monomer interacts with the C-terminal domain of the opposing monomer (Figure 5; Table 1). This style of interaction is generally conserved among enzymes of the DSOR family. For example in glutathione reductase, His467, located near the *C*-terminus of one monomer, is necessary for catalytic function of the opposing monomer (Misra et al., 1985). In MerA, this has been substituted to a catalytically important tyrosine (Rennex et al., 1994).

Structural superimposition of *Mse*MerA (described here) with the recently solved Tn*501*MerA structure with bound mercury (4K7Z) reveals two specific amino acid substitutions, V317 to E, and Y441′ to F′, in the active site of *Mse*MerA compared to Tn*501*MerA (numbering is by Tn*501*MerA 4K7Z) (Figure 5). Another residue thought to be involved in metal coordination, Y100 (in *Bc* structure is Y264) (Schiering et al., 1991), is strictly conserved. For Tn*501*MerA and *Bc*MerA, the hydroxyl groups of Y441′ and Y100 likely act in concert to facilitate metal transfer from the C-terminal cysteines to the active site cysteines. In contrast, in *Mse*MerA, the F441′ in the position of tyrosine in Tn*501*MerA lacks a hydroxyl group to coordinate the Hg^2+^ ion, but a glutamic acid in place of the Tn*501*MerA V317 provides a different residue with which the Hg^2+^ ion could potentially be coordinated.

The conservation of either the V/Y′ in mesophiles or the E/F′ amino acid pair in thermophiles, along with the observed positions of the amino acids, is suggestive of an alternative metal binding strategy for Hg^2+^ ion transfer from the C′ cysteine pair to the active site cysteines C42 and C47. In Tn*501*MerA and *Bc*MerA, upon Hg^2+^ ion binding to the C′ cysteines, the C′ terminal region folds into the catalytic cleft, delivering the mercuric ion (Lian et al., 2014) to the conserved Y100 and Y441′, which facilitate transfer to the active site cysteines. Given that *Mse*MerA lacks the Y441 with which to coordinate the Hg^2+^ion during active site delivery, the E317 is the most rational alternative.

Rennex et al. (1994) have previously substituted individual amino acids Y441F and Y100F in *Bc*MerA. The *K*_m_ for Hg^2+^ increased from 30 to 39 μM in the case of the Y441F variant, and decreased to 6 μM in the case of the Y100F variant. However, in both cases, the *K*_cat_/*K*_m_ was decreased around 15-fold. It is therefore likely that the observed low catalytic efficiency of the variant enzymes is due in part to a lack of a residue to coordinate the Hg^2+^ ion, such as the glutamic acid found in *Mse*MerA and other thermophiles. Moreover, Sayed et al. (2013) previously demonstrated that glutamic acid residues may play a role in Hg^2+^ ion coordination and transfer. However, the active site glutamic acid found in *Mse*MerA is a different site from what Sayed et al. (2013) have previously characterized. Furthermore, sequence alignment shows that the ATII-LCL enzyme has the V/Y amino acid pair (Table 1).

Both the Tn*501*MerA Y441′ and the *Mse*MerA E300 are about 5 Å from the active site cysteines, although they coordinate from different positions, with the Y441′ coordinating the Hg^2+^ ion almost perpendicular to E317. The different placement and nature of these side chains may help explain the higher *K*_m_ observed in *Mse*MerA relative to homologs from mesophilic organisms. Since the high Hg^2+^ concentrations are common features of high temperature environments, these differences may reflect adaptations to function at elevated Hg^2+^ concentrations and as such represent the structural determinants of specificity for mercuric reductases. Highly specific stable enzymes, especially those that catalyze oxidation-reduction reactions coupled to the specific molecular recognition, could potentially be used as chemical sensors in which the redox chemistry could be coupled to produce an amplifiable electrical signal.

In conclusion, here we present a characterization of the thermostable mercuric reductase from *M. sedula*. We show that the enzyme is highly resistant to heat treatment while retaining similar catalytic rates to other characterized MerAs. The enzyme appears to have a potentially different way of coordinating Hg^2+^ and has a lower affinity for Hg^2+^ ions than previously characterized enzymes. Considering that *Mse* is a thermophile and its MerA is likely to harbor properties more similar to those of primitive MerA that evolved in a high temperature environments (Barkay et al., 2010), these results may indicate that the activity of MerA has been refined through evolutionary time to successfully detoxify environmental Hg^2+^ at lower concentrations than those that are naturally present in thermal environments.

## Conflict of Interest Statement

The authors declare that the research was conducted in the absence of any commercial or financial relationships that could be construed as a potential conflict of interest.

## Supplementary Material

The Supplementary Material for this article can be found online at http://journal.frontiersin.org/article/10.3389/fbioe.2015.00097
