# Supplementary material for: Biochemical and Structural Properties of a Thermostable Mercuric Ion Reductase from Metallosphaera sedula
Source: Front Bioeng Biotechnol. 2015 Jul 13;3:97. doi: 10.3389/fbioe.2015.00097 (PMC4500099; doi:10.3389/fbioe.2015.00097)
Supplement: Supplementary file 1 [file Data_Sheet_1.DOCX]

*Mse*MerA sequence used: MGSSHHHHHHSQDPMHKLAIIGYGAAGFAAMIKANELGVKPVLIGKGEIGGTCVNVGCVPSKRMLYIAEIYKKAREVTGSEVYPPFSSFQEKDGLVQEMRKTKYEDLLSYYDVELIQGEARFISPHAVKVNGQVIEAEKFVIATGSSPLIPRIPGLDKVGFWTNREALSPDRRIDSLAVIGGRALALEFAQMYSRMKVEVAILQRSPVLIPDWEPEASVEARRIMENDGVAVVTGVNVKEVRKGAGKIVITDKGEVEADEILLATGRKPNVDLGLENAGVRLNERGGIKVDDELRTDNPHIYAAGDVLGGKMLEALAGRQGSIATENALTGSHKRVDENAVPQVIFTQPNLARVGLTEAEARAKEGEVEARVLPMSSVAKAEIINSRLGFVKMVTMNGRIVGVHAVGENVAEMIGEAALAIRFGATVHDLIDTVHMFPTIAESLRLVALAFRSDVSRLSCCV
