# Supplementary figures and images for: Biochemical and Structural Properties of a Thermostable Mercuric Ion Reductase from Metallosphaera sedula
Source: Front Bioeng Biotechnol. 2015 Jul 13;3:97. doi: 10.3389/fbioe.2015.00097 (PMC4500099; doi:10.3389/fbioe.2015.00097)

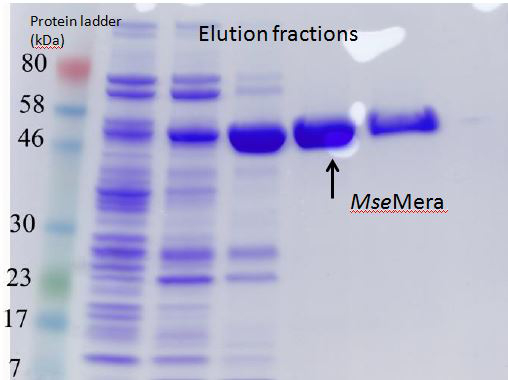

Supplement: Supplementary file 2 [file Image_1.JPEG]

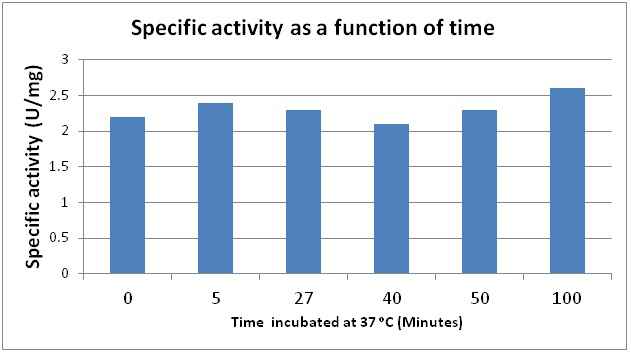

Supplement: Supplementary file 3 [file Image_2.JPEG]

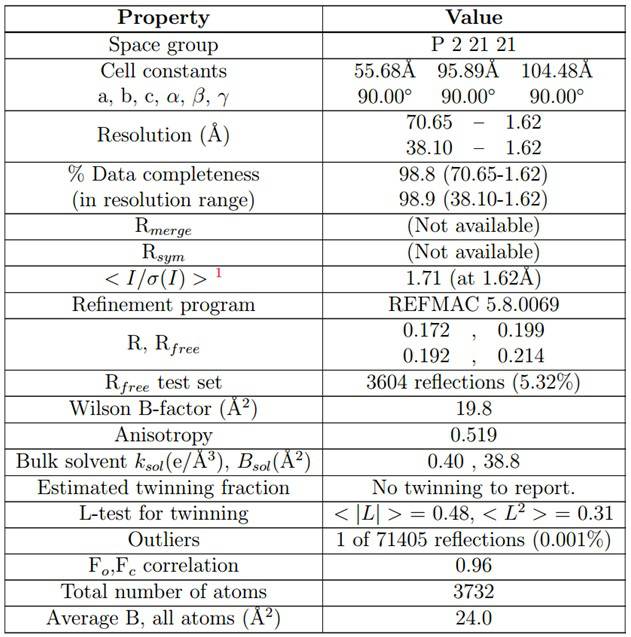

Supplement: Supplementary file 4 [file Image_3.JPEG]
